# Relative Validity of MijnEetmeter: A Food Diary App for Self-Monitoring of Dietary Intake

**DOI:** 10.3390/nu13041135

**Published:** 2021-03-30

**Authors:** Marga Ocké, Ceciel Dinnissen, Annette Stafleu, Jeanne de Vries, Caroline van Rossum

**Affiliations:** 1 National Institute for Public Health and the Environment (RIVM), 3720 BA Bilthoven, The Netherlands; ceciel.dinnissen@rivm.nl (C.D.); caroline.van.rossum@rivm.nl (C.v.R.); 2 Division of Human Nutrition and Health, Wageningen University and Research, 6708 WE Wageningen, The Netherlands; jeanne.devries@wur.nl; 3 Netherlands Nutrition Centre, 2508 CK The Hague, The Netherlands; stafleu@voedingscentrum.nl

**Keywords:** relative validity, food record, 24-h dietary recall, app, web-based, dietary feedback

## Abstract

This study aimed to evaluate the relative validity of intake of energy, nutrients and food groups assessed with MijnEetmeter food diary as compared to 24-h dietary recalls, and if this differed between experienced and new users. One hundred men and women aged 18–70 y participated, of whom 47 had prior experience with the tool. Participants kept MijnEetmeter on three days. Trained dietitians called them three times for a 24-h dietary recall interview, once recalling food consumption on the same day as the food recording in MijnEetmeter. Systematic differences and correlations were assessed, and Bland–Altman plots were created; both for 3-day mean intakes and for intakes on the same day. Relative to 24-h dietary recalls, MijnEetmeter underestimated consumption of drinks, added fat, cereal products, and potatoes. Relative underestimation was observed for energy intake (6%) and about half of the nutrients. Experienced MijnEetmeter users underestimated intake the least. For intake of energy and six key nutrients, correlations between 3-day mean intakes were above 0.7 except for sodium intake. In conclusion, MijnEetmeter moderately underestimates intakes of energy and some nutrients and food groups. To improve the self-monitoring of dietary intake, it is recommended that the users record food consumption for several days and that the apps probes for easily forgotten foods and drinks.

## 1. Introduction

Diet is an important modifiable risk factor for being overweight, as well as prevalent non-communicable diseases, such as cardiovascular disease, diabetes mellitus, and several types of cancer [1]. Various advisory bodies, therefore, advocate to limit energy intake and to shift the dietary pattern towards diets rich in plant-based foods, such as vegetables, fruit, legumes, and nuts, and with limited intake of sugar-sweetened beverages, meat, salt, and (saturated) fats [2,3].

Dietary counseling is traditionally given by dietitians, and is primarily aimed at persons at elevated risk for or having a diet-related illness [4,5]. Recently, a large number of applications have become available for self-monitoring dietary intake; the applications provide dietary feedback tailored to users’ dietary intake and personal dietary goals [6,7]. Self-monitoring supports individuals to become aware of their dietary pattern and has been associated with weight loss [8]. Moreover, dietary feedback that fits an individuals’ personal goal and dietary pattern has been shown to be more effective than general advice [6]. Self-monitoring tools are of interest because of their relatively low costs and their potential use by many consumers who can access them throughout the day and anywhere [9]. Recent reviews concluded that many users find those tools attractive, but that few of them have been validated [6,9].

In 2009, the Netherlands Nutrition Centre launched the application ‘MijnEetmeter’ for dietary tracking and tailored dietary feedback. In 2019, the app/webtool had 19 million visits per year. The aim of this study was to determine how well MijnEetmeter is able to assess the daily intake of energy, nutrients, and food groups in comparison with dietitian-administered 24-h dietary recalls. Interest was primary in systematic differences at group level, and secondary in the ability to rank users by intake, and relative validity at the individual level. Moreover, the study aimed to assess whether relative validity of MijnEetmeter differed between new users versus experienced users.

## 2. Materials and Methods

Based on a power calculation, the aim was to collect complete data on 100 participants, half with- and half without prior experience in using MijnEetmeter. In order to account for potential drop-out, 120 persons were recruited. Participants with prior experience in using MijnEetmeter for self-monitoring of their diet were recruited on the website of MijnEetmeter, while participants without prior experience were recruited from a consumer panel of Kantar Public Netherlands. Having prior experience with MijnEetmeter was defined as having used MijnEetmeter at least 5 times before study recruitment, while no prior experiences was defined as never used MijnEetmeter before study recruitment. Potential participants were eligible if they were aged 18 to 70 years and able to use MijnEetmeter on their smartphone, tablet, or computer. Exclusion criteria were not speaking the Dutch language, being pregnant, breastfeeding, being a professional or student in dietetics or nutrition, and participating in other research at the same time.

The Medical Ethical Review Committee of Utrecht University evaluated that the study was not subject to the Medical Research Involving Human Subjects Act (WMO) of the Netherlands (METC protocol 19-184/C). Medical-ethical review was, thus, not needed. All study participants provided written informed consent. 

With MijnEetmeter, a person can keep a food diary on any day and receives feedback about the composition of his or her diet. The application can be used online (www.voedingscentrum.nl/mijneetmeter) or as an app available in the Google Play store or Apple App store. In MijnEetmeter, foods can be searched through text searching. The smart-phone version has the extra option to scan the barcode of food packages. The food list includes over 90,000 foods, based on both the generic Dutch Food Composition database [10] and the Dutch Branded Food database [11]. In addition, new foods and their composition can be added by the user. A recipe function is available, but mixed dishes can also be chosen from the food list. Consumed food amounts can be indicated via various options that always include the weight in grams or volume in ml, and often the number or fraction of household measures or natural or commercial units. 

MijnEetmeter calculates the daily consumption of food groups, according to the food classification of the Dutch Wheel of Five food based dietary guidelines. The five components are ‘vegetables and fruits’, ‘spreadable and cooking fats’, ‘dairy, nuts, fish, legumes, meat and eggs’, ‘bread, grain/cereal products, and potatoes’, and ‘drinks’ [12]. Moreover, MijnEetmeter shows the calculated daily intake of energy, fat, saturated fat, carbohydrates, protein, fiber, and salt and, optionally, 22 other nutrients. Each branded food is linked to a comparable food in the generic Dutch food composition database [10], to allow for calculating intake of nutrients not listed on the label. MijnEetmeter shows its users the percentage of energy intake contributed by foods that fit in the Wheel of Five. For foods outside the Wheel of Five, alternative foods with the same role in the dietary pattern are suggested (e.g., brown bread instead of white bread). Moreover, MijnEetmeter shows per food group how much was consumed and what the advised consumption is. It provides tips how to improve consumption of this food group and recipe suggestions. The app also indicates whether the consumption of nutrients is in accordance with the Dutch reference values. 

The 24-h dietary recalls were administered through telephone interviews using the online software Compl-eat [13]. The software started with a quick list, in which the participant was asked to list roughly all consumed foods for 6 potential eating occasions. In a second step, the interviewer asked questions to specify each food. Quantification was done in terms of gram, ml, or commonly consumed commercial, natural, or household units. Mixed dishes entered as new or standard recipes were disaggregated into their ingredients.

Data were collected in the autumn of 2019. After completing an online questionnaire, participants kept a food diary in MijnEetmeter on three days, and completed three 24-h dietary recalls by trained dietitians. The online questionnaire collected information on participant’s general, anthropometric and lifestyle characteristics. Each method, MijnEetmeter and the 24-h dietary recall, was administrated three times on non-consecutive days which included one weekend-day and two weekdays. Half of the participants started with MijnEetmeter and the other half with the 24-h dietary recall. The 24-h dietary recall after the first MijnEetmeter food diary referred to the same day. Consequently, for the participants who started with MijnEetmeter, the first 24-h recall was about the same day as MijnEetmeter, and for participants who started with a 24-h dietary recall, the second recall was about the same day as MijnEetmeter. For all other administrations there were at least 4 days in between the food diary days and the recalled days. For the 24-h recall that referred to the same day as the first MijnEetmeter food diary, an appointment was made, whereas the other 24-h recalls were unannounced unless three contact attempts were without success. 

All foods reported in the 24-h dietary recalls were categorized into the food groups mentioned in the Wheel of Five food based dietary guidelines [12]. MijnEetmeter already classified foods according to this classification. Few products, such as salt and herbs, sweeteners, sugar, cacao powder, and special dieting products, such as meal replacers, were classified as miscellaneous and not considered in the present statistical analyses. For both methods, consumption of food groups per person per day and, subsequently, the 3-day average consumptions were calculated. Intakes of energy and nutrients per person per day were exported from MijnEetmeter and Compl-eat tools; both used the same food composition database [10]. Three-day mean intakes of energy and nutrients were calculated for both methods for each participant. Dietary supplements were not considered in this study. All extreme high values in energy, nutrient and food group intakes in the 24-h recalls were evaluated, using cut-of levels derived from the Dutch national food consumption survey [14]. The foods that contributed the most to the high intakes were checked for obvious errors, such as incorrect choices of portion size options. The amounts were changed for less than 10 foods, e.g., from 150 portions to 150 g. No data were excluded based on the outlier evaluation.

Frequency analyses were conducted to describe the study population in terms of socio-demographic characteristics and lifestyle. For the total population and for the groups with and without prior experience with MijnEetmeter, the medians and interquartile ranges of the 3-day mean food group consumption assessed by both methods were calculated. Because of skewed distributions, the non-parametric Wilcoxon’s signed rank test was used to test if the distribution of food group consumption assessed with MijnEetmeter and with 24-h dietary recalls differed systematically. Moreover, Spearman’s rank correlation coefficients were calculated between the 3-day mean intakes assessed with both methods, and between the 1-day intakes that referred to the same consumption day.

For intake of energy, macronutrients and micronutrients assessed with both methods, means, and standard deviations were calculated for the total population and stratified by prior experience with MijnEetmeter. A paired *t*-test was conducted to test if there was a systematic difference between both methods; mean differences and their 95% confidence intervals were derived. Pearson’s correlation coefficients were calculated between the 3-day average nutrient intakes assessed with both methods, and between the 1-day intakes that referred to the same consumption day. Exceptions were vitamins A and B_2_ for which Spearman correlation coefficients were calculated because of a few extremely high outlier values in MijnEetmeter data. Bland–Altman plots were made, plotting the mean intake assessed with both methods against the difference in intake. The derived 95% limits of agreement [15] are presented to provide information on the variation in individual relative validity.

All statistical analyses were conducted using SAS version 9.4 (SAS Institute Inc., Cary, NC, USA). *p*-values < 0.05 were considered statistically significant, and statistical testing was done two-sided.

## 3. Results

### 3.1. Study Population Characteristics

Of the 120 recruited participant, 20 dropped out of the study before the first dietary assessment. A total of 100 adults, of which 64 were women, completed three days of MijnEetmeter and three 24-h dietary recalls (Table 1). In the study population, the prevalences of age up to 50 years, a high educational level (higher vocational education or university), and a body mass index between 18.5 and 25 kg/m^2^ were just over 50%. Almost half of the study population (47%) had experience with MijnEetmeter prior to the study. The experienced group consisted of more women (72% vs. 57%), more participants with a higher educational level (62% vs. 43%), and more participants who did not drink alcohol (51% vs. 32%) than the inexperienced group. In addition, the experienced group included more participants with a weight loss of 3 kg or more in the last 6 months (40% vs. 19%), as well as a specific dietary regimen (26% vs. 13%) or following a special diet (36% vs. 13%).

### 3.2. Relative Validity for Food Groups

For most food groups, the consumption distributions assessed with MijnEetmeter and with 24-h recalls did not differ significantly (see Table 2). Statistically significant differences between the two methods were only observed for the consumption of potatoes (medians 43 vs. 58 g/day), drinks (medians 1373 vs. 1685 g/day), cereal products (medians 30 vs. 47 g/day), mixed dishes (medians 37 vs. 0 g/day), and added fats (medians 8 vs. 16 g/day). Thus, with exception of the mixed dishes, MijnEetmeter underestimated intake of those food groups compared to the 24-h dietary recalls.

Based on these results, some additional analyses were conducted. For drinks, the lower median consumption assessed with MijnEetmeter was also observed for non-energy containing drinks (1070 vs. 1470 g/day, respectively), whereas this was not the case for energy-containing drinks (169 vs. 160 g/day). Persons who reported at least 50 g/day higher consumption of mixed dishes in MijnEetmeter as compared to the 24-h dietary recalls, had a lower consumption of main components of dinner. Median differences were 46 g for potatoes, 22 g for cereal products, 64 g for vegetables, and 32 g for meat. These results are consistent with the breaking down of mixed dishes into ingredients in the 24-h dietary recall data handling, rather than misreporting of mixed dish intake by the participants.

Spearman correlations between consumption of food groups averaged over three days assessed with MijnEetmeter and 24-h dietary recalls ranged from 0.14 (mixed dishes) to 0.81 (milk and milk products), with a median correlation of 0.57. For most food groups, correlations for consumption assessed for the same day were higher and ranged from 0.35 (mixed dishes) to 0.94 (fish), with a median of 0.80.

Only the consumption of mixed dishes differed significantly between the two methods in both the groups with and without prior experience with MijnEetmeter; see Supplemental Online Material Table S1. Specific in persons without prior MijnEetmeter experience, the median consumptions of added fats, cereal products, and drinks, assessed with MijnEetmeter (7, 18, and 1142 g/day, respectively), were significantly lower than consumptions assessed with the 24-h dietary recalls (20, 43, and 1642 g/day, respectively). The group with prior experience in MijnEetmeter entered a lower consumption of the food group mixed dishes in MijnEetmeter (median value 0 g/day) than the group without prior experience (median value 67 g/day).

Spearman correlation coefficients for the consumption of food groups assessed with MijnEetmeter and 24-h dietary recalls (three-day averages) ranged from 0.04 (mixed dishes) to 0.85 (snacks) with a median of 0.58 in the participants with prior MijnEetmeter experience. In the participants without prior experience, it ranged from 0.18 (mixed dishes) to 0.78 (milk and milk products), with a median of 0.52.

In Table S2 of the Supplemental Online Material, relative validity for food groups is tabled by gender. Among women, statistical significant differences between methods were observed for the same food groups except for potatoes as in the total population. For the small group of 35 men, only the differences for added fats and mixed dishes were statistically significant. See Supplemental Material. Median correlation coefficients were 0.59 and 0.80 for 3-day and the same day comparison among women, and 0.48 and 0.78 for 3-day and the same day comparison among men.

### 3.3. Relative Validity for Nutrients

The mean intakes of energy, total and saturated fat, carbohydrates, and mono- and disaccharides expressed in g/day, and total fat expressed as a percentage of energy intake were significantly lower as assessed by MijnEetmeter than in the 24-h dietary recalls. The intakes of protein and sodium, and saturated fat, protein, carbohydrates, and mono- and disaccharides expressed as a percentage of energy intake did not differ significantly (Table 3). For energy intake, the difference was 6% lower; the differences for intakes of total fat, saturated fatty acids, and mono- and disaccharides were, with 8–10%, the largest. Pearson correlation coefficients between intakes assessed with MijnEetmeter and 24-h dietary recalls ranged for the nutrients in Table 3 from 0.40 for fat as percentage of energy intake to 0.86 for mono- and disaccharides. For the overlapping day, correlations ranged from 0.36 for fat as percentage of energy intake to 0.82 for mono- and disaccharides. Particularly, for sodium, the correlation was higher when intakes of the same day (0.75) were compared versus intakes averaged over three days (0.47).

**Figure 1:**
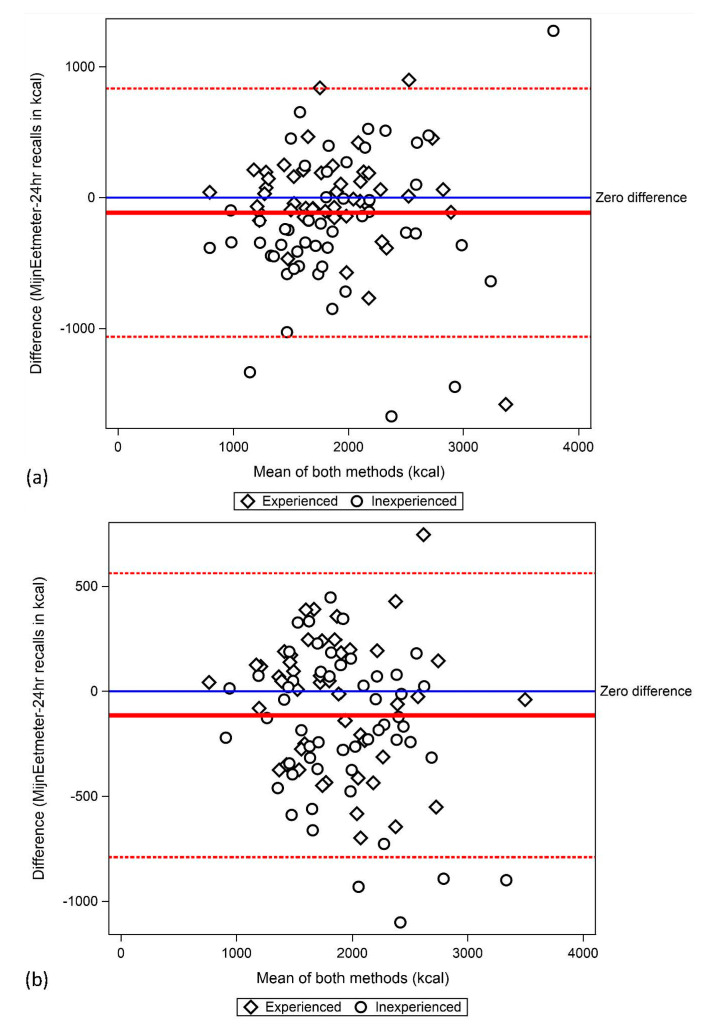
Bland–Altman plot for energy intake assessed with MijnEetmeter and 24-h dietary recalls. (**a**) Results based on one single overlapping day. (**b**) Results based on 3-day means of each method.

Figure 1 shows Bland–Altman plots for energy intake. Based on the intake of the overlapping day, the mean difference in energy intake was 114 kcal, with 95% limits of agreement from −1062 to 835 kcal. Based on three-day mean intakes, the mean difference in energy intake was the same, but the 95% limits of agreement (LOA) were narrower (−789 to 562 kcal). For other nutrients, 95% LOA based on three-day mean intakes are all wide (Table 3). 

Table S3 of the Supplementary Online Material shows mean intakes for 22 additional nutrients that MijnEetmeter users can optionally ask for. For 9 out of the 22 nutrients, the mean intake assessed by MijnEetmeter was significantly lower than in the 24-h dietary recalls. The highest significant differences were observed for water (16%) and vitamin E (11%). Correlation coefficients between 3-day average intakes of MijnEetmeter and the 24-h dietary recalls ranged from 0.39 for selenium to 0.90 for iodine, with a median correlation coefficient of 0.68. For intakes on the overlapping intake day, a similar range was observed.

Within the participants with prior experience with MijnEetmeter, differences in intake of energy and the six nutrients in the standard output were not statistically significant (Table 4). In the inexperienced group, however, intake differences of MijnEetmeter and the 24-h dietary recalls were significant for energy (177 kcal), fat (9 g), carbohydrates (19 g), and mono- and disaccharides (10 g), with lower intakes in MijnEetmeter. 

Based on the differences between experienced and inexperienced users, additional analyses regarding the relative validity of energy intake for subgroups in the population were conducted. See Supplementary Online Material Table S4. The subgroups were chosen based on characteristics for which the experienced and the unexperienced participants differed. Both men and women had a lower mean energy intake assessed with MijnEetmeter as compared to the 24-h dietary recalls; the mean difference was larger in men (163 kcal vs. 81 kcal). A significant difference was also observed in persons not following a special diet and persons that did not lose 3 kg or more body weight during the last six months.

## 4. Discussion

In this study, dietary intake as assessed with the MijnEetmeter tool was compared with intake collected through interview-based 24-h dietary recalls as reference method. For the food groups drinks, cooking fat, cereal products, and potatoes, MijnEetmeter underestimated consumption relative to the 24-h dietary recalls. This translated into underestimations of water, fats, vitamin E, and, to a lesser extent, energy and various other nutrients, such as intake of total fat, saturated fatty acids, carbohydrates, and mono- and disaccharides. In the group of experienced MijnEetmeter users, underestimation by MijnEetmeter was smaller for most food groups and nutrients than in the group of inexperienced users. 

In addition, in other studies, energy and total fat intake was underestimated by food diary apps in comparison to interviewer-assisted 24-h dietary recalls [16,17,18,19], though the differences were not always statistically significant [20,21,22,23]. Underestimation was generally smaller for protein intake and, to a lesser extent, carbohydrate intake [24]. Relative validity for micronutrients and food groups is not often reported [24] and, thus, cannot be compared well. 

The relative validity for MijnEetmeter at the individual level varied a lot. For energy intake, for 95% of the participants, the difference between MijnEetmeter and 24-h dietary recalls was expected to fall between about −1100 and +800 kcal/day. In a recent review, the observed average range was similar, i.e., 1918 kcal [24]. This indicates that it is extremely difficult to obtain similar results for each individual using two different dietary assessment methods. The correlations between MijnEetmeter and the 24-h dietary recalls were between 0.5 and 0.8 for most food groups and nutrients, which is comparable with similar studies [24]. However, since not only true intake but also error might be correlated between the methods, it is expected that the correlation coefficients between true intake and intake assessed with MijnEetmeter are lower [25].

The observed higher intake of mixed dishes in MijnEetmeter data can be explained by a difference in food classifications in MijnEetmeter and the 24-h dietary recalls data. In MijnEetmeter, mixed dishes were considered as a food, whereas, in the Compl-eat software for 24-h dietary recalls, most mixed dishes were split into their ingredients. In addition, other studies have shown the considerable impact of mixed dishes disaggregation [26,27]. Not disaggregating mixed dishes in MijnEetmeter data may affect the correctness of the dietary feedback about the food groups. The group with prior experience with MijnEetmeter reported less mixed dishes in the app compared to the inexperienced group. Although it is possible that the experienced group really consumed less mixed dishes, it is likely that they used the recipe function of MijnEetmeter more often, so foods were in the database in their disaggregated form.

In line with our results on underestimation of added fats, the validity study of e-CA (electronic carnet alimentaire, ‘food record’ in French) observed that foods, such as cooking fats, were often forgotten in the app, whereas the dietitians specifically probed for these, which resulted in an observed higher intake [17]. A possible explanation for the underreporting of beverages in the MijnEetmeter is that participants might have considered non-energy drinks irrelevant to record. This is consistent with the finding that underestimation was not observed for energy-containing drinks. The specific underreporting of cooking fats and drinks calls for considering inclusion of specific reminder pop-ups or probes for these two food groups in MijnEetmeter. Such probes are common practice as part of multiple-pass 24-h recall software [28,29]. However, adding probes to food diary apps needs to be considered also in the light of participant burden and irritation. Focus groups conducted for MyFood24 24-h dietary recall learned that participants preferred a straightforward, less complicated tool [30].

Several strengths and limitations should be addressed. This is one of few studies [21,31,32] that reports relative validity of a self-administered food record tool for more than 10 food groups or nutrients among a study sample of more than 50 persons. The performance of MijnEetmeter was assessed relative to that of dietitian-administered 24-h dietary recalls. It is well-known that this reference method is not a gold standard [33], so true validity of MijnEetmeter remains unknown. However, comparison to a dietitian-administered 24-h dietary recall is relevant since the MijnEetmeter tool could be positioned as a compromise between general one-fit-for-all dietary advice, and personal dietetic counseling. Designing a relative validation study is challenging, since various unwanted effects, such as learning and memory effects and differences due to day-to-day variation in intake, might occur [25]. We tried to tackle those challenges in the study design. A limitation was that the study population might not have been fully representative of the usual MijnEetmeter users, although half of them were recruited through the MijnEetmeter website. On the other hand, the study design allowed to differentiate results for experienced and inexperienced Eetmeter users, which, to our knowledge, has not been reported before and provided important insights suggesting learning effects. The experienced and inexperienced group did, however, differ in background characteristics, such as gender, being on a diet, or having lost weight, and likely also in motivation, awareness of his/her eating pattern, and perceived ability to self-monitor dietary intake. These factors might have partly explained the observed differences. A last strength to mention is that we described MijnEetmeter and reported its relative validation according to best practice guidelines [9]. Because MijnEetmeter aims to support consumers to eat healthier, it is advised to evaluate the tool regarding this ultimate aim, too.

## 5. Conclusions

In conclusion, the relative validity of energy and macronutrient intake assessed with MijnEetmeter tool was similar to that of other self-administered apps. However, there appears to be more underreporting in MijnEetmeter than in interviewer-administered 24-h recalls. Underreporting was less severe in the experienced MijnEetmeter users, and there was high interperson variability in relative validity. Development of instruction videos or additional functionalities that stimulate complete food recording, especially for beverages and cooking fats, whilst limiting participant burden are recommended. In order to improve the tailored dietary feedback, it is also recommended that mixed dishes are split into ingredients in the data handling of MijnEetmeter. Moreover, each participant should be encouraged to record several days of food consumption as experience improves accuracy.

## Figures and Tables

**Table 1:** Participant characteristics in MijnEetmeter-study (*n* = 100).

Variables	Total Population	Experience with MijnEetmeter		No Experience with MijnEetmeter	
	%	*n* (47)	%	*n* (53)	%
Gender					
Male	35	13	28	22	42
Female	64	34	72	30	57
Other	1	0	0	1	2
Age category (years)					
20–50	51	23	49	28	53
51–70	49	24	51	25	47
Highest educational level attained					
Low ^1^	15	6	13	9	17
Middle ^2^	31	11	23	20	38
High ^3^	52	29	62	23	43
Unknown	2	1	2	1	2
Smoking status					
Never or previous smoker	90	40	85	50	94
Current smoker	10	7	15	3	6
Alcohol consumption frequency					
None	41	24	51	17	32
1 day or less per week	36	12	26	24	45
2 or more days per week	23	11	23	12	23
BMI category					
BMI < 18.5 kg/m^2^	1	1	2	0	0
18.5 ≤ BMI ≤ 25 kg/m^2^	55	27	57	28	53
BMI > 25 kg/m^2^	44	19	40	25	47

^1^ low educational level; primary education, lower vocational education, advanced elementary education; ^2^ middle educational level; intermediate vocational education, higher secondary education; ^3^ high educational level; higher vocational education and university.

**Table 2:** The 50th, 25th, and 75th percentile of consumption of food groups (g/day) as assessed with MijnEetmeter and with 24-h dietary recalls and their correlation in MijnEetmeter Study (*n* = 100).

Food Groups	MijnEetmeter 3-Day Average			24-h Dietary Recalls 3-Day Average			Difference	Spearman Correlation Coefficient	
	P50	P25	P75	P50	P25	P75	*p*-Value *	3-Day Means	Same Day
Vegetables	179	72	248	182	123	286	0.23	0.61	0.68
Fruit	160	79	243	184	96	245	0.49	0.79	0.88
Added fats	8	0	17	16	9	26	<0.001	0.43	0.39
Fish	0	0	23	0	0	33	0.80	0.52	0.94
Legumes	0	0	0	0	0	0	0.85	0.40	0.57
Meat	64	40	94	77	43	116	0.12	0.64	0.81
Eggs	17	0	33	11	0	33	0.95	0.53	0.74
Nuts	8	0	20	8	0	21	0.82	0.65	0.85
Milk and milk products	233	117	359	245	134	392	0.48	0.81	0.89
Cheese	20	10	40	32	13	48	0.05	0.61	0.80
Bread	108	82	152	119	87	172	0.13	0.57	0.89
Cereal products	30	12	64	47	19	90	0.02	0.33	0.72
Potatoes	43	0	88	58	24	111	0.02	0.35	0.82
Drinks	1373	900	1722	1685	1294	2054	<0.001	0.64	0.63
Sandwich spreads	8	0	22	10	2	19	0.80	0.68	0.82
Soups	0	0	83	0	0	81	0.93	0.48	0.86
Snacks	45	18	84	59	23	93	0.28	0.68	0.80
Sauces	8	0	25	10	0	28	0.40	0.29	0.43
Mixed dishes	37	0	156	0	0	5	<0.001	0.14	0.35

* Wilcoxon signed rank test (normal approximation) testing the differences between 3-day average intakes of MijnEetmeter and the 24-h dietary recalls.

**Table 3:** Means (SD), differences, and correlation coefficients for energy and nutrient intakes as assessed with ‘MijnEetmeter’ and with 24-h dietary recall in all participants in MijnEetmeter Study (*n* = 100) *.

Nutrients	MijnEetmeter		24-h Dietary Recalls		Difference			Bland–Altman 95% LOA		Pearson’ s Correlation Coefficient	
	Mean	SD	Mean	SD	Mean	95% CI		Lower	Upper	3-Day Means	Same Day
Energy (kcal)	1830	485	1944	549	−114	−181	−47	−789	562	0.79	0.69
Fat (g)	70	24	77	27	−7	−11	−4	−47	32	0.71	0.61
Fat (En%)	33.0	9.0	35.0	7.0	−3.0	−5.0	−1.0	−21.0	15.0	0.40	0.36
Saturated Fatty Acids (g)	25	10	27	11	−2	−4	−1	−17	13	0.75	0.67
Saturated Fatty Acids (En%)	11.0	4.0	12.0	4.0	−1.0	−2.0	0.0	−8.0	6.0	0.59	0.51
Protein (g)	77	26	79	24	−2	−5	1	−35	31	0.79	0.77
Protein (En%)	16.0	5.0	17.0	4.0	0.0	−1.0	1.0	−8.0	7.0	0.73	0.77
Carbohydrates (g)	199	61	209	67	−9	−18	−1	−91	72	0.80	0.76
Carbohydrates (En%)	42.0	10.0	43.0	7.0	−1.0	−3.0	0.0	−16.0	14.0	0.66	0.64
Mono– and disaccharides (g)	80	31	87	34	−7	−10	−3	−42	29	0.86	0.82
Mono– and disaccharides (En%)	17.0	6.0	18.0	5.0	−1.0	−2.0	0.0	−9.0	6.0	0.77	0.78
Sodium (mg)	2307	909	2254	854	54	−126	234	−1762	1869	0.47	0.75

* Results are based in 3-day mean intakes unless otherwise indicated: LOA = limits of agreement; CI = confidence interval; En% = percentage of energy intake.

**Table 4:** Mean and differences in energy and nutrient intakes as assessed with ‘MijnEetmeter’ and with 24-h dietary recalls in experienced (*n* = 47) and inexperienced ‘MijnEetmeter’ users (*n* = 53) in MijnEetmeter Study *.

Nutrients	Experienced MijnEetmeter Users					Inexperienced MijnEetmeter Users				
	MijnEetmeter	24-h Dietary Recalls	Difference			MijnEetmeter	24-h Dietary Recalls	Difference		
	Mean	Mean	Mean	95% CI		Mean	Mean	Mean	95% CI	
Energy (kcal)	1831	1874	−42	−135	50	1829	2006	−177	−272	−81
Fat (g)	66	72	−5	−11	0	73	82	−9	−15	−4
Saturated Fatty Acids (g)	22	24	−2	−4	0	27	30	−3	−5	0
Protein (g)	80	82	−1	−7	4	74	77	−3	−7	1
Carbohydrates (g)	204	203	1	−10	12	196	214	−19	−30	−7
Mono – and disaccharides (g)	81	83	−2	−7	2	80	90	−10	−15	−5
Sodium (mg)	2205	2184	21	−214	256	2398	2315	83	−193	358

* based on mean intakes over 3 days MijnEetmeter and three 24-h dietary recall; CI = confidence interval.

## Data Availability

The data presented in this study are available on request from the corresponding author. The data are not publicly available due to comply with GDPR requirements of the General Data Protection Regulation (EU) 2016/679.

## Supplementary Materials

The following are available online at https://www.mdpi.com/article/10.3390/nu13041135/s1, Table S1: Median consumption of food groups (g/day) as assessed with 3-day MijnEetmeter and with three 24-h dietary recalls and their correlation, in persons with and without prior experience with MijnEetmeter in the MijnEetmeter Study, Table S2. The 50th, 25th, and 75th percentile of consumption of food groups (g/day) as assessed with MijnEetmeter and with 24-h dietary recalls and their correlation in MijnEetmeter Study, by gender. Table S3. Mean intake and (standard deviation) of nutrients as assessed with three day MijnEetmeter and with three 24-h dietary recalls and their difference and correlation, in the Eetmeter Study. Table S4. Mean energy intake in kcal/day and (standard deviation) as assessed with three-day MijnEetmeter and with three 24-h dietary recalls and their difference, for subgroups in the MijnEetmeter Study (*n* = 100).
