# Supplementary material for: Relative Validity of MijnEetmeter: A Food Diary App for Self-Monitoring of Dietary Intake
Source: Nutrients. 2021 Mar 30;13(4):1135. doi: 10.3390/nu13041135 (PMC8066644; doi:10.3390/nu13041135)
Supplement: Supplementary file 1 [file nutrients-13-01135-s001.pdf]

Table S1. Median consumption of food groups (g/day) as assessed with 3-day MijnEetmeter and with three 24-h dietary recalls and their correlation, in persons with and without prior experience with MijnEetmeter in the Eetmeter Study.

	Experienced Users (*n* = 47)				Inexperienced Users (*n* = 53)			
**Food Group**	**MijnEetmeter**	**24-h Dietary Recalls**	***p*-Value ***	**Spearman Correlation Coefficient**	**MijnEetmeter**	**24-h Dietary Recalls**	***p*-Value ***	**Spearman Correlation Coefficient**
Vegetables	217	205	0.78	0.69	103	165	0.06	0.57
Fruit	193	204	0.88	0.83	128	150	0.43	0.76
Added fats	10	13	0.17	0.76	7	20	<0.001	0.24
Fish	0	0	0.37	0.47	0	0	0.67	0.57
Legumes	0	0	0.78	0.30	0	0	0.75	0.26
Meat	63	71	0.90	0.72	65	79	0.05	0.56
Egg	17	15	0.65	0.58	1	8	0.61	0.51
Nuts	13	12	0.83	0.70	7	5	0.82	0.58
Milk and milk products	259	261	0.66	0.81	200	223	0.59	0.78
Cheese	22	31	0.16	0.63	20	32	0.18	0.59
Bread	106	113	0.67	0.47	108	122	0.10	0.62
Cereal products	30	50	0.19	0.46	18	43	<0.001	0.20
Potatoes	33	53	0.05	0.16	47	70	0.21	0.52
Drinks	1583	1700	0.09	0.67	1142	1642	<0.001	0.62
Sandwich spead	8	10	0.62	0.65	8	10	0.88	0.71
Soups	1	2	0.90	0.43	0	0	0.78	0.49
Snacks	33	36	0.64	0.85	60	71	0.38	0.52
Sauces	9	8	0.85	0.37	8	14	0.16	0.29
Ready-made meals	0	0	<0.001	0.04	67	0	<0.001	0.18

* Wilcoxon sign rank test, normal approximation.

Table S2. The 50th, 25th and 75th percentile of consumption of food groups (g/day) as assessed with MijnEetmeter and with 24-h dietary recalls and their correlation in MijnEetmeter Study, by gender (*n* = 100).

Food Group	*n*	MijnEetmeter			24hr Dietary Recalls			Wilkoxon*	Spearman Correlation Coefficients	
		P50	P25	P75	P50	P25	P75	*p*-Value*	3-Day Means	Same Day
					Males					
Vegetables	35	117	63	222	153	104	218	0.36	0.57	0.62
Fruit	35	183	88	278	210	113	256	0.75	0.77	0.93
Added fats	35	7	0	15	20	11	37	0.00	0.49	0.28
Fish	35	0	0	33	0	0	33	0.60	0.49	0.91
Legumes	35	0	0	0	0	0	0	0.92	0.39	0.47
Meat	35	90	67	118	108	58	143	0.41	0.41	0.67
Eggs	35	0	0	17	4	0	33	0.48	0.45	0.83
Nuts	35	18	0	27	15	0	30	0.95	0.56	0.81
Milk and milk products	35	223	120	342	245	143	392	0.51	0.77	0.89
Cheese	35	20	10	38	32	12	47	0.12	0.43	0.78
Bread	35	135	92	175	167	96	208	0.24	0.48	0.89
Cereal products	35	50	0	100	88	33	129	0.14	0.25	0.83
Potatoes	35	33	7	67	47	10	103	0.29	0.21	0.68
Drinks	35	1417	967	1700	1638	1146	2047	0.10	0.83	0.80
Sandwich spreads	35	7	0	20	13	3	20	0.47	0.69	0.88
Soups	35	0	0	83	0	0	69	0.36	0.30	0.77
Snacks	35	63	35	95	81	41	119	0.29	0.51	0.68
Sauces	35	9	0	26	13	0	25	0.75	0.23	0.30
Mixed dishes	35	117	0	243	0	0	107	0.01	0.02	0.53
					Females					
Vegetables	64	199	107	278	201	153	313	0.42	0.63	0.72
Fruit	64	155	77	230	161	96	238	0.56	0.78	0.84
Added fats	64	8	1	18	14	8	23	0.00	0.43	0.51
Fish	64	0	0	7	0	0	32	0.43	0.54	0.97
Legumes	64	0	0	0	0	0	0	0.87	0.39	0.71
Meat	64	59	26	82	66	33	99	0.15	0.62	0.87
Eggs	64	17	0	33	16	0	33	0.66	0.55	0.73
Nuts	64	7	0	17	6	0	17	0.76	0.69	0.86
Milk and milk products	64	242	111	378	247	132	400	0.74	0.83	0.87
Cheese	64	23	10	42	31	12	48	0.30	0.71	0.82
Bread	64	104	82	129	113	82	143	0.24	0.59	0.87
Cereal products	64	33	0	83	50	4	97	0.08	0.37	0.80
Potatoes	64	28	13	58	49	21	84	0.02	0.42	0.71
Drinks	64	1327	871	1750	1722	1317	2078	0.00	0.56	0.57
Sandwich spreads	64	9	1	22	10	2	18	0.83	0.69	0.79
Soups	64	0	0	83	0	0	83	0.58	0.59	0.89
Snacks	64	34	14	81	40	17	81	0.68	0.69	0.83
Sauces	64	8	0	21	10	1	28	0.28	0.37	0.49
Mixed dishes	64	22	0	138	0	0	0	0.00	0.15	0.11

* Wilcoxon signed rank test (normal approximation) testing the differences between 3-day average intakes of MijnEetmeter and the 24hr dietary recalls.

Table S3. Mean intake and (standard deviation) of nutrients as assessed with three day MijnEetmeter and with three 24-h dietary recalls and their difference and correlation, in the MijnEetmeter Study (n = 100).

**Nutrient**	**Eetmeter**		**24-h Dietary Recalls**		**Difference**				**Pearsons Correlation Coefficient**	
	**Mean**	**SD**	**Mean**	**SD**	**Mean**	**95% Confidence Interval**		**% (Compared to 24hr Recall)**	**3-Days**	**1 Day**
Vegetable protein (g)	32.8	11.4	34.7	12.4	−1.9	−3.6	−0.3	−6	0.75	0.76
Fibre (g)	24.2	10.3	24.5	8.8	−0.3	−1.5	0.9	−1	0.81	0.76
Alcohol (g)	6	11	6	11	0	−1	1	2	0.79	0.79
Water (g)	2214	745	2644	720	−430	−542	−318	−16	0.70	0.67
Vitamin A (ug)	888	1,890	692	355	196	−189	581	28	0.41*	0.59
Vitamin D (ug)	2.4	1.5	2.8	1.8	−0.4	−0.7	0.0	−14	0.42	0.59
Vitamin E (mg)	10.9	4.3	12.2	4.7	−1.3	−2.2	−0.5	−11	0.51	0.54
Vitamin B1(mg)	0.93	0.33	1.01	0.34	−0.08	−0.13	−0.02	−7	0.63	0.52
Vitamin B2(mg)	3.70	23.35	1.42	0.59	2.27	−2.36	6.90	160	0.69*	0.66
Vitamin B6 (mg)	1.37	0.49	1.50	0.44	−0.13	−0.20	−0.05	−8	0.68	0.64
Folate (folic acid equivalents) (ug)	258	101	274	94	−16	−31	0	−6	0.68	0.70
Vitamin B12 (ug)	3.98	3.01	4.13	3.15	−0.16	−0.83	0.52	−4	0.40	0.52
Vitamin B3 (mg)	15.7	6.7	16.5	6.0	−0.8	−2.0	0.4	−5	0.54	0.60
Vitamin C (mg)	97	68	96	52	1	−9	10	1	0.71	0.79
Potassium (mg)	3137	1044	3297	951	−159	−277	−42	−5	0.83	0.73
Calcium (mg)	904	324	992	373	−88	−140	−37	−9	0.73	0.70
Magnesium (mg)	336	119	365	123	−30	−45	−14	−8	0.79	0.79
Iron (mg)	10.9	3.5	11.1	3.4	−0.2	−0.9	0.5	−2	0.49	0.34
Selenium (ug)	49	26	49	24	0	−5	6	0	0.39	0.81
Zinc (mg)	9.5	3.0	9.8	2.8	−0.3	−0.8	0.1	−3	0.67	0.70
Iodine (ug)	184	145	189	232	−5	−29	19	−3	0.90	0.93
Phosphorus (mg)	1311	405	1428	421	−117	−170	−64	−8	0.79	0.70

* correlation coefficients are Spearman correlations because of extreme values in MijnEetmeter data

Table S4. Mean energy intake in kcal/day and (standard deviation) as assessed with three day MijnEetmeter and with three 24-h dietary recalls and their difference, for subgroups in the MijnEetmeter Study (n = 100).

**Subgroup**	***n***	**MijnEetmeter**		**24hr Dietary Recalls**		**Difference**	
		**Mean**	**SD**	**Mean**	**SD**	**Mean**	**95% CI**
Gender							
Men	35	2165	504	2183	504	−163	−293−33
Women	64	1658	364	1642	364	−81	−159 −3
Following a special diet							
Yes	24	1615	444	1533	444	−58	−163 47
No	76	1898	480	1872	480	−131	−214−49
Weight loss of 3 kg or more in the last 6 months							
Yes	29	1796	471	1796	471	−19	−155116
No	51	1939	497	1961	497	−131	−222−39
